# Imatinib-induced podocytopathies in a patient with gastrointestinal stromal tumor

**DOI:** 10.1080/0886022X.2021.1930049

**Published:** 2021-05-26

**Authors:** Gang Wang, Ning Zhuo, Ying Luo, Jie Li

**Affiliations:** a Department of Rheumatology and Immunology, Zhuzhou Hospital Affiliated to Xiangya Medical College, Central South University, Zhuzhou, Hunan, China; b Department of Nephrology, Second Xiangya Hospital, Central South University, Changsha, Hunan, China; c Department of Nephrology, Zhuzhou Hospital Affiliated to Xiangya Medical College, Central South University, Zhuzhou, Hunan, China

Dear Editor,

A 52-year-old Chinese male presented to the nephrology department in December 2017 with generalized swelling of the face and extremities and skin peeling. He had no previous history of hypertension, diabetes, or chronic kidney disease and was not taking any potentially nephrotoxic medications. He underwent gastrointestinal stromal tumor (GIST) resection in general surgery due to abdominal pain 2 months before. Immunohistochemistry revealed that Vim (+), Pan-CK (−), CD34 (+), S100 (−), HHF35 (−), SMA (−), CD117 (+), Dog-1 (+), SDHB (+), P53 (−), EMA (+), Ki67 index 10%. The histology results showed that gastrointestinal stromal tumors affected the entire thickness of the gastric wall, but there was no tumor metastasis in the perigastric lymph nodes (0/1) (Figure 1). To prevent tumor recurrence and improve overall survival, thus, he was treated with imatinib 400 mg/day after the operation. However, while the patient was taking imatinib for 2 months, he developed generalized swelling of the face and extremities and skin peeling. The nephrologist considered that the only drug the patient was taking during this period was imatinib and discontinued it. Noticeably, his renal function and blood pressure were normal before oral imatinib. Negative for 24-h urine protein, and serum creatinine 86 μmol/L. Currently, his pulse rate was 89 beats per minute and his blood pressure was 104/68 mmHg, both of which fluctuated within normal range. He had no tumor lysis syndrome and no signs of dehydration. Laboratory tests showed that 24-h urine protein 9.3 g/d; serum albumin 21 g/L; serum creatinine 522 μmol/L; negative for ANCA and anti-GBM antibodies; IgG 4.07 g/L; hyperlipidemia; total protein 41.7 g/L; albumin 25.6 g/L; globulin 15.8 g/L; Cl 111.7 mmol/L; Ca 1.83 mmol/L; CA-199 13.8 U/ml; AFP and CEA were normal. Abdominal computed tomography showed postoperative changes of the gastrointestinal stromal tumor. Renal ultrasound showed diffuse parenchymal lesions in both kidneys. The size of the left kidney is about 117 × 60 mm, and the size of the right kidney is about 117 × 48 mm. He was initially diagnosed with nephrotic syndrome based on proteinuria, hypoproteinemia, and hyperlipidemia. Electron microscopy revealed diffuse podocyte foot process effacement, which was considered as podocytopathies (minimal change disease or underlying focal segmental glomerulosclerosis) with acute tubulointerstitial injury. Immunofluorescence showing no immune complex deposition. Eventually, the diagnosis of imatinib-induced podocytopathies was given to this patient. Imatinib was immediately stopped and oral glucocorticoid 1 mg/kg QD was administered. After a regular follow-up for one year, his renal function recovered and serum creatinine fluctuated within the normal range, but there was still a little urinary protein (Figure 2). From 2019 to 2021, the 24-h urine protein was negative along with normal renal function.

**Figure 1.**
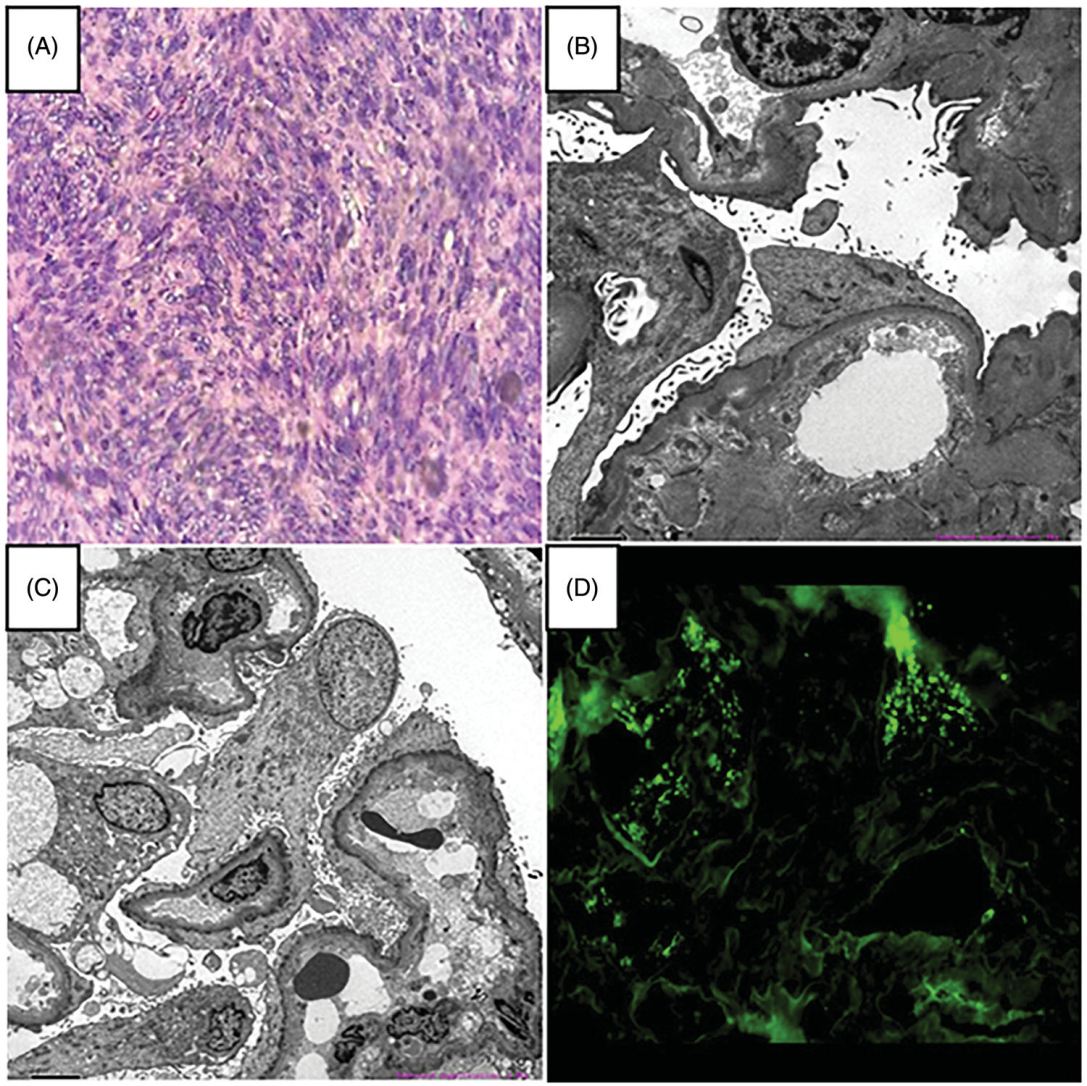
(A) Histological examination of the gastrointestinal stromal tumor (H&E staining × 200). (B, C) Electron microscopy revealed diffuse podocyte foot process effacement, which was considered as podocytopathies (minimal change disease or underlying focal segmental glomerulosclerosis) with acute tubulointerstitial injury (magnification × 2000). (D) Immunofluorescence showing no immune complex deposition (IgG-A-M, C3, C1q).

**Figure 2.**
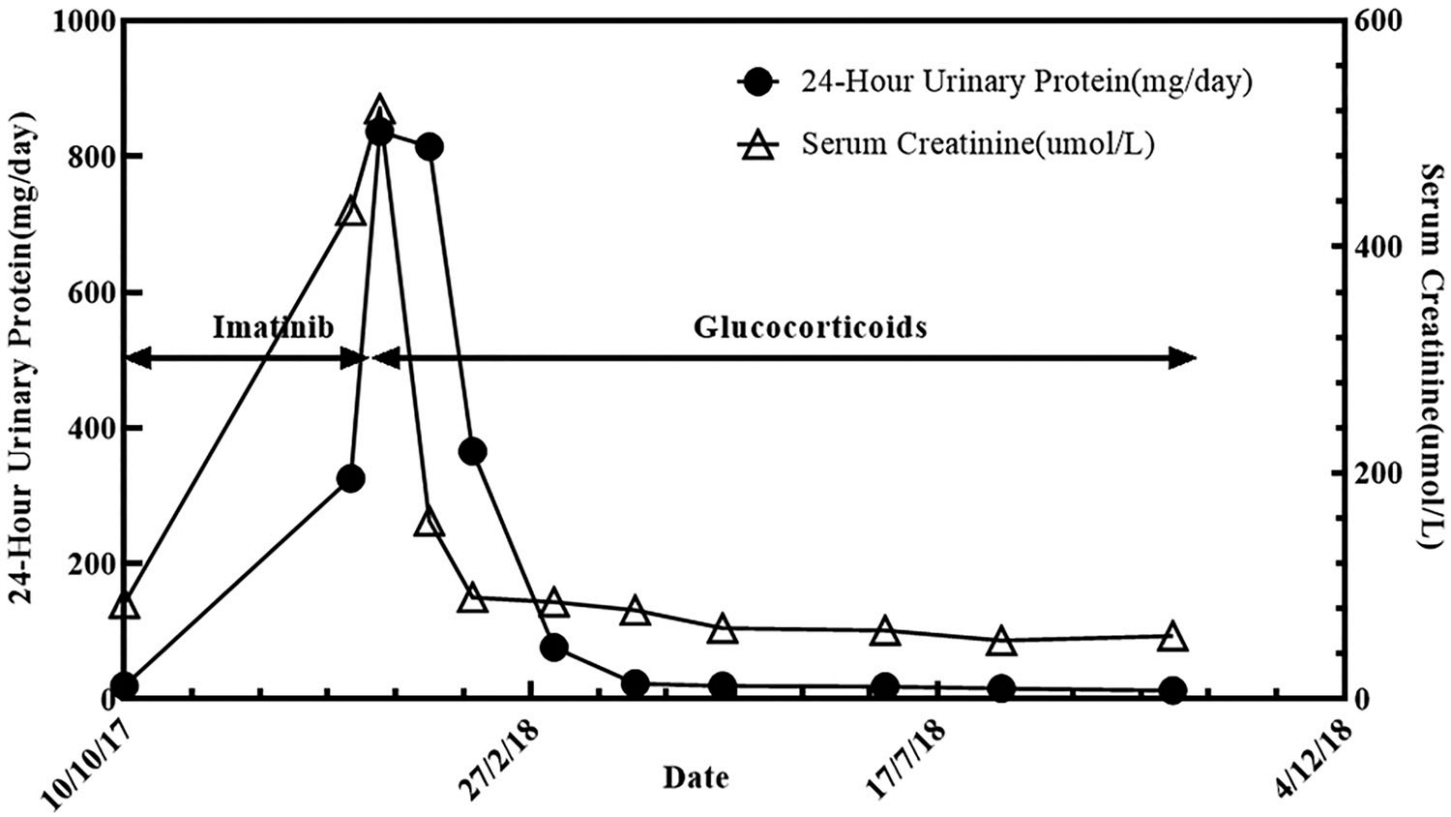
Significant increase in proteinuria and creatinine during imatinib treatment (arrows). The rapid decrease in proteinuria and creatinine after the stop of imatinib and use of glucocorticoids (arrows).

As a tyrosine kinase inhibitor, imatinib is widely used in the treatment of chronic myeloid leukemia and GIST [1]. Studies by Yilmaz et al. [2] and Chen et al. [3] both showed that acute kidney injury (AKI) occurs in patients with chronic myeloid leukemia treated with tyrosine kinase inhibitors, with imatinib being more common. The exact mechanism by which imatinib causes AKI is unclear, and acute tubular necrosis (ATN) may be the major cause [4]. The proliferation and regeneration of renal proximal tubular cells depend on the activation of PDGFR, and imatinib inhibits PDGFR and blocks PDGF activation, resulting the inability of renal proximal tubular cells to proliferate and regenerate [4]. Apoptosis of renal proximal tubular cells blocks the renal tubules and eventually leads to acute tubular necrosis. However, glomerular podocyte changes caused by imatinib is very rare. How imatinib causes podocytopathies and its pathogenesis draws our attention.

Podocytes are terminally differentiated cells located outside the glomerular basement membrane, and the slit membrane formed by podocytes is the final barrier for glomerular filtration [5]. After podocyte injury, the structural integrity of the podocyte is impaired, the podocyte process disappears, the pore diameter of the filtration membrane increases or breaks, macromolecular proteins are filtered out, and proteinuria is formed beyond the reabsorption capacity of the proximal renal tubule. Nephrin protein and Fyn are the basis of podocyte structure and function, and play a joint role in maintaining podocyte structure and morphology, regulating function, and activating signaling pathways (Figure 3) [6,7]. Normally, podocytes have vascular endothelial growth factor (VEGF) production. After the injury, podocytes lose this production, it is the reason for endothelial cell injury. Receptor tyrosine kinase (RTK) is a superfamily, VEGFR belongs to it. RTKI such as imatinib can cause nephrotic syndrome (minimal change disease or focal segmental glomerulosclerosis) by overexpression of c-mip in podocytes because of inactivation of RelA, while c-mip is undetectable in healthy humans [8]. In animal studies, only when the podocytes of the transgenic mouse kidney express c-mip, can the diffuse effacement of the foot process of podocytes be observed, confirming that c-mip is the main protein that leads to pathological changes [9]. The high expression of c-mip in podocytes blocks the Fyn-Nephrin protein signaling pathway, preventing podocytes from participating in normal filtration and secretion of VEGF [9]. Thus, both c-mip overexpression and RelA inactivation have an important role in pathogenesis. Close monitoring of renal function and early detection of potential renal damage from imatinib is vital to improving the long-term prognosis of patients.

**Figure 3.**
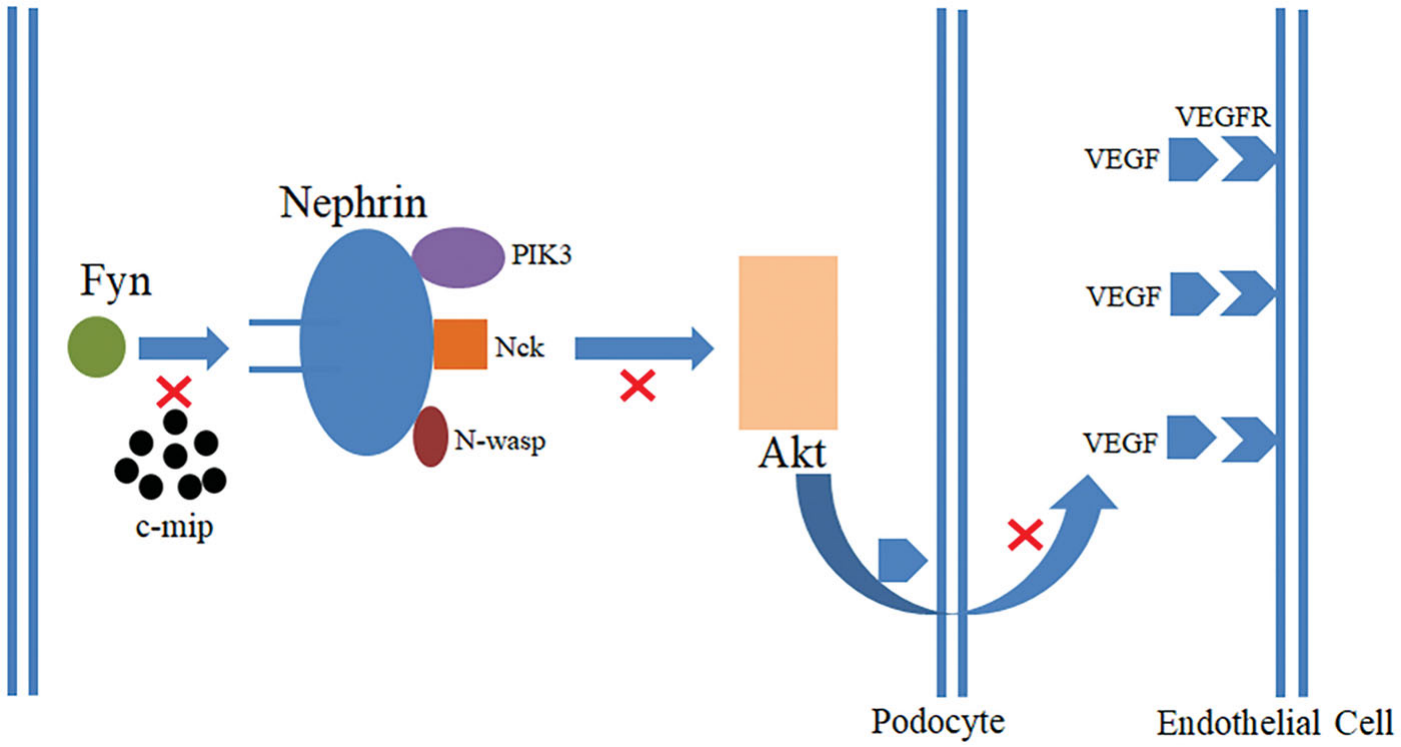
The high expression of c-mip in podocytes blocks the Fyn-Nephrin protein signaling pathway, preventing Nephrin from recruiting PIK3, NcK, and N-wasp. Nephrin is unable to phosphorylate and activate Akt, and podocytes cannot participate in normal filtration barrier and secretion of vascular endothelial growth factor (VEGF).

## Data Availability

All data are included in the manuscript.
